# SOCS3 Expression by Thymic Stromal Cells Is Required for Normal T Cell Development

**DOI:** 10.3389/fimmu.2021.642173

**Published:** 2021-03-18

**Authors:** Yu Gao, Ruining Liu, Chenfei He, Juan Basile, Mattias Vesterlund, Marie Wahren-Herlenius, Alexander Espinoza, Cassandra Hokka-Zakrisson, Fahad Zadjali, Akihiko Yoshimura, Mikael Karlsson, Berit Carow, Martin E. Rottenberg

**Affiliations:** ^1^Department of Microbiology, Tumor and Cell Biology, Karolinska Institutet, Stockholm, Sweden; ^2^SciLife Lab, Department of Oncology-Patohology, Karolinska Institutet, Stockholm, Sweden; ^3^Department of Medicine, Karolinska Institutet, Stockholm, Sweden; ^4^Broegelmann Research Laboratory, Department of Clinical Science, University of Bergen, Bergen, Norway; ^5^College of Medicine and Health Sciences, Sultan Qaboos University, Muscat, Oman; ^6^Department of Microbiology and Immunology, Keio University School of Medicine, Tokyo, Japan

**Keywords:** SOCS3, thymus, T cells, thymic epithelial cell, TRIM21

## Abstract

The suppressor of cytokine signaling 3 (SOCS3) is a major regulator of immune responses and inflammation as it negatively regulates cytokine signaling. Here, the role of SOCS3 in thymic T cell formation was studied in *Socs3*^*fl*/*fl*^ *Actin-creER* mice (Δ*socs3)* with a tamoxifen inducible and ubiquitous *Socs3* deficiency. Δ*socs3* thymi showed a 90% loss of cellularity and altered cortico-medullary organization. Thymocyte differentiation and proliferation was impaired at the early double negative (CD4-CD8-) cell stage and apoptosis was increased during the double positive (CD4+CD8+) cell stage, resulting in the reduction of recent thymic emigrants in peripheral organs. Using bone marrow chimeras, transplanting thymic organoids and using mice deficient of SOCS3 in thymocytes we found that expression in thymic stromal cells rather than in thymocytes was critical for T cell development. We found that SOCS3 in thymic epithelial cells (TECs) binds to the E3 ubiquitin ligase TRIM 21 and that *Trim21*^−/−^ mice showed increased thymic cellularity. Δ*socs3* TECs showed alterations in the expression of genes involved in positive and negative selection and lympho-stromal interactions. SOCS3-dependent signal inhibition of the common gp130 subunit of the IL-6 receptor family was redundant for T cell formation. Together, SOCS3 expression in thymic stroma cells is critical for T cell development and for maintenance of thymus architecture.

## Introduction

The function of the thymus is to generate T lymphocytes that express T cell receptors with sufficient diversity to combat different microorganisms and tumors, while eradicating potentially autoreactive T cells. The thymus is histologically structured into discrete peripheral cortical and central medullary regions. These regions contain distinct stromal cell populations where thymic epithelial cells (TECs) are the main cell type, as well as immature T cells referred as thymocytes at defined stages of maturation. Diverse subsets of TECs in the cortex (cTECs) and medulla (mTECs) provide signals required for the survival and differentiation of thymocytes. The stepwise progression of thymocyte development requires their migration through these thymic regions, where interactions with cTEC and mTEC subsets take place (1).

Thymocytes enter the thymus as CD4-CD8- double negative (DN) progenitors. DN cells are subdivided into 4 sequential stages (DN1-DN4), based on the expression of CD44 and CD25. TCRγ and TCRδ rearrangements are completed at the DN3 stage when TCRβ rearrangement start. Progression beyond the DN3 stage requires expression of TCRβ, and TCRα chain rearrangement follows. Thymocytes expressing TCRαβ upregulate CD4 and CD8 [entering the double positive (DP) stage] and are further selected by cTECs to become CD4 or CD8 single positive (SP) cells binding either MHC-I or MHC-II molecules with their TCR (1, 2). After further negative selection in the medulla, SP thymocytes leave the thymus as functional mature T cells (3).

Cytokines are essential for the coordination of the stepwise T cell development in the thymus. Some cytokines, such as IL-7, are produced by TECs, support the proliferation and survival of thymocytes (4–6). Also, cytokines produced by thymocytes stimulate proliferation and differentiation of TECs (7–9).

A tight control of cytokine release and responses to cytokines is required for the correct development of T cells. The suppressor of cytokine signaling-3 (SOCS3) hampers signaling in response to the IL-6 family of cytokines. SOCS3 binds to the common gp130 subunit of the IL-6 receptor family impairing STAT3 activation (10). SOCS3 also regulates responses to cytokines, growth factors, and hormones that are independent of gp130 [i.e., IL-12R, granulocyte-colony stimulation factor (G-CSF), leptin, insulin] (11). SOCS3 is a central regulator of immunity and of the differentiation of diverse lymphoid and myeloid populations (12). Of importance, SOCS3 has been also been shown to regulate B cell lymphopoiesis, granulopoiesis, and erythropoiesis (13–15).

STAT3-mediated signaling has been demonstrated to contribute to optimal development of mTECs (but not cTECs) (16, 17). Comparatively little is known about the function of SOCS3 during thymic T cell development. Studies so far suggest that SOCS3 has a limited role during early thymopoiesis *in vitro* (18, 19).

Given the importance of SOCS3 in regulating different stages in T cell and the importance of the thymus in T cell maturation and homeostasis, the role of SOCS3 in T cell differentiation in the thymus was analyzed in this study. Since the genetic deletion of SOCS3 leads to mid-gestational embryonic lethality (13, 20), *Socs3*^*fl*/*fl*^ *actin-creER* mice (Δ*socs3*) showing an inducible and tissue-broad deletion of *Socs3* were used in this study. Our results show a critical role of SOCS3 in T cell formation in the thymus and in the maintenance of thymic cellularity and architecture, mediated by the regulation of thymic stromal functions.

## Materials and Methods

### Mice

The animals were housed according to directives and guidelines of the Swedish Board of Agriculture, the Swedish Animal Protection Agency, and the Karolinska Institute (djurskyddslagen 1988:534; djurskyddsförordningen 1988:539; djurskyddsmyndigheten DFS 2004:4). The study was performed under approval of the Stockholm North Ethical Committee on Animal Experiments permit number N397/13 and N3506/17.

Mice were housed at the Dept. of Microbiology, Tumor and Cell Biology the Astrid Fagreus and the Wallenberg Laboratories, Karolinska Institutet, Stockholm, Sweden, under specific pathogen-free conditions.

Mice containing loxP-flanked socs3 alleles have been described before (21). To allow temporal control of Cre activity, mice transgenic for a fusion between Cre and a mutated ligand-binding domain of the estrogen receptor (CreERT2) under the control of the β-actin promoter (CAGGCre-ER^TM^) (22) were crossed with *Socs3*^*fl*/*fl*^ mice (21) are referred as Δ*socs3* mice.

For a lymphoid-specific deletion *Socs3*^*fl*/*fl*^ were bred with *lck cre* (23) and *cd4 cre* transgenic animals (24). *Gp130*^*F*/*F*^ mice with an aminoacid substitution within gp130 abrogating the SOCS3 binding site have been described before (25). *Trim21*^−/−^ mice were generated by homologous recombination as previously described (26).

The C57BL/6 congenic strain carrying the differential pan leukocyte marker CD45.1 was used in bone marrow radiation chimeric mice studies.

### Flow Cytometry

Single cell suspensions from spleen, lymph node (LN) and thymus were obtained by mechanical disruption, straining over a 40-μm nylon mesh and lysis of erythrocytes. Cells were counted and surface stained with respective antibodies: anti-CD3 (clone: 17A2), anti-CD4 (GK1.5), anti-CD8 (53-6.7), anti-CD44 (IM7), anti-CD62L (MEL-14), anti-CD127 (A7R34), anti-CD24 (M1/69), anti-Qa-2 (695H1-9-9), anti-βTCR (H57-597), anti-γδTCR (GL3) all from eBioscience), and anti-CD25 (7D4) from BD Pharmingen). For analysis of thymic cell populations a dump channel with markers of lineage positive cells including CD11b (monocytes), Ter19 (erythrocytes), Ly6G (neutrophils), CD19/B220 (B cells), NK1.1 (NK cells) was included.

For characterization of TECs, a previously described thymic stromal cell isolation procedure was used (27). Thymi were dissected from freshly killed mice and trimmed of fat and connective tissue. Small cuts into the capsules were made with a pair of fine scissors and the thymi were gently agitated in 50 ml of RPMI-1640 with a magnetic stirrer at 4°C for 30 min to remove the majority of thymocytes. The resulting thymic fragments were transferred into 10 ml of fresh RPMI-1640 and dispersed further to free more thymocytes. The thymic fragments were then incubated in 5 ml of 0.125% (w/v) collagenase D with 0.1% (w/v) DNAse I (both from Boehringer Mannheim, Germany) in RPMI-1640 at 37°C for 15 min, with gentle agitation. Enzyme mixtures with isolated cells were removed after fragments had settled, then replaced with fresh mixture for further incubation. Gentle mechanical agitation was performed with a 3-ml syringe and 26G needle to break up aggregates remaining in final digestions. After 2 digestions, cells were centrifuged, resuspended in 5 mM EDTA in PBS+1% FCS+0.02% (w/v) NaN_3_ (EDTA/FACS buffer) and allowed to incubate for 10 min at 4°C to disrupt rosettes. Cells were then passed through 100-μm mesh to remove clumps, and cells were labeled with antibodies as described above.

Single cell suspension was stained with CD45 (clone 30-F11, eBioscience), EpCAM (clone G8.8, eBioscience), Ly51 (clone 6C3, BD Pharmingen), UEA-1 (Vector Laboratories), CD80 and MHCII antibodies.

Apoptosis was determined by Annexin-V binding according to supplier's protocol (BD Pharmingen). Data were acquired in LSRII flow cytometer (BD) and analyzed using FlowJo software (Tree star).

### Thymus Transplantation

The survival surgery was performed under sterile conditions after intra-peritoneal administration of the anesthetics, ketamine (100 mg/kg) and xylazine (10 mg/ kg) to CD45.1+ mice as described (28). A small dorsolateral incision was made to expose the kidney and a small hole was made in the kidney capsule. One fifth of a thymic lobe from 1 week old WT or Δ*socs3* (CD45.2+) donors were placed under the kidney capsule and the incision was closed with sterile sutures. One month after the transplantation, recipient mice were treated with Tm for 5 days. The graft dissected for flow cytometric analysis 7 days after the last Tm dose. The grafted thymus was analyzed for CD45.1 (derived from recipients) and CD45.2 (carried over from grafted thymus) cells.

### BrdU Incorporation

WT and Δ*socs3* mice were injected intraperitoneally with 5-bromo-2-deoxyuridine (BrdU; Sigma; 0.1 mg/g) and were sacrificed 4 or 72 h after injection. Mice were sacrificed 10 days after Tm administration. For FACS analysis, single-cell suspensions were prepared from the thymi of BrdU pulse-labeled mice. Thymocytes were incubated with CD4, CD8, IL-7R, αβ, and γδ TCR antibodies followed by BrdU staining using the FITC BrdU Flow Kit (BD Pharmingen).

### TEC Sorting

Thymic stroma were separated after enzyme digestion as described above. Then CD45^neg^ cells were negatively selected using MACS magnetic beads labeled with anti-CD45 antibodies following instructions from the manufacturer. Cells were further labeled with anti-CD45 and anti-EpCAM antibodies and selected EpCAM+ cells sorted using a FACSAria^TM^ Fusion device.

### Overexpression of SOCS3

Transfection of CMV-driven SOCS3 EGFP expressing constructs, empty vector control and GFP-expressing plasmid was performed with lipofectamine following the indications of the manufacturer. In brief OP9-DL1 cells in 50–60% confluent in 100 mm dishes, were washed and incubated in 1.5 ml serum free OptiMEM and transfected with 14 μg plasmid and 5 μl.

Lipofectamine 3000, for 8 h 37°C. Cells then were washed and incubated in 11 ml OptiMEM + 10% fetal calf serum, mercaptoethanol for 12 h 37°C. Cells were then washed and incubated with OptiMEM 10% FCS at 32°C for 24 h. Then, cells were lysed for subsequent WB or IP studies. The efficiency of transfection was also analyzed by FACS.

### Immunoprecipitation

Transfected and control OP9-DL1 cells 1.5 × 10^7^ were resuspended in lysis buffer (120 mM NaCl, 50 mM Tris pH 8.0, 0.5% NP-40 and protease inhibitor cocktail p8340, Sigma), incubated for 1 h and then centrifuged at 14,000 × g for 10 min. Supernatants (1 mg protein/ ml) were incubated with 1 μg mouse anti-Myc (clone 9E10, Santa Cruz Biotechnology) or isotype antibodies overnight at 4°C. Samples were incubated then with Protein-G Agarose (Santa Cruz Biotechnologies) 4 h at 4°C. The samples were then washed 3 times in PBS 0.1% Tween and frozen for subsequent LC/MS-MS analysis or resuspended in 20 ul Laemli buffer, boiled for 5 min for Western blot.

### Western Blot

Soluble protein concentration from OP9-DL1 cells was quantified by DC™ Protein Assay Kit (5000111; Bio-Rad). Thirty microgram of total protein were then mixed with 4x Laemmli buffer containing 8% SDS and β-mercaptoethanol followed by heat-denaturation for 5 min and cooled 15 min RT. Immunoprecipitated proteins or lysed proteins were separated by electrophoresis (Invitrogen NuPAGE electrophoresis system) through 4–12% Bis-Tris gradient gels with MOPS running buffer and transfered to a nitrocellulose membrane. The NC membranes were then blocked in PBS 5% BSA 0.1% Tween and incubated with primary mouse anti-Myc tag (Santa Cruz), rabbit anti-TRIM-21 or mouse anti-GAPDH antibodies (In vitrogen, H 06737 and 6C5) overnight at 4°C. NC membranes were then washed and incubated with HRP-conjugated anti-rabbit or anti-mouse IgGs at RT for 1 h. The membranes were developed using enhanced chemiluminescence (ECL, GE Health Care).

### Mass Spectrometry

On-bead reduction, alkylation and digestion (trypsin, sequencing grade modified, Pierce) was performed followed by SP3 peptide clean-up of the resulting supernatant (29). Each sample was separated using a Thermo Scientific Dionex nano LC-system in a 3 h 5–40% ACN gradient coupled to Thermo Scientific High Field QExactive. The software Proteome Discoverer vs. 1.4 including Sequest-Percolator for improved identification was used to search the mouse Uniprot database for protein identification, limited to a false discovery rate of 1%.

### Microarray Analysis

Total RNA was isolated from WT and Δ*socs3* TECs using the RNeasy Total RNA Isolation Kit (Qiagen) and cRNA was prepared as described. Briefly, cDNA was specifically transcribed from 500 ng of mRNA using a Poly-T nucleotide primer containing a T7 RNA polymerase promoter (Geneworks). Biotinylated, antisense target cRNA was subsequently synthesized by *in vitro* transcription using the BioArray High Yield RNA Transcript Labeling kit (Enzo Diagnostics). Fifteen micrograms of biotin-labeled target cRNA was then fragmented and used to prepare a hybridization mixture, which included probe array controls and blocking agents. Hybridization, washing and scanning were performed according to the manufacturers recommendations. Absolute levels of expression of genes were determined and scaled to 150 using algorithms in MicroArray Analysis suite 5.0 (MAS5) software (Affimetrix). The signal value represents the level of expression of a transcript and was expressed as a log_2_ ratio. In-depth analyses and clustering of data were conducted using GeneSpring software (Silicon Genetics). Normalization was performed using a per chip 50th percentile normalization and per gene median normalization method. Genes that were consistently absent or below the noise level were excluded from analysis.

To identify genes with statistically significant differences between WT and Δ*socs3* TECs a *p*-value cut-off of 0.05 and the Benjamini and Hochberg false discovery rate as multiple testing correction were performed. The Student-Newman-Keuls *post-hoc* test was used to identify the specific groups in which significant differential expression occurred. Genes that showed a change of 2-fold or greater were considered differentially expressed. The IPA and WebGEstalt softwares were used to identify pathways and gene sets based on common functional features that are differentially expressed in Δ*socs3* TECs. The raw and processed data has been deposited in the GEO public repository with the series accession number GSE165216.

### Histopathology

Thymi, lungs and livers from Δ*socs3* and WT mice were processed for histological analysis. In brief, organs were fixed in 4% buffered paraformaldehyde for 24 h and paraffin-embedded. Eight micrometer sections were obtained, paraffin-removed and dehydrated before staining with hematoxylin and eosin (Sigma-Aldrich).

### Immunohistochemistry

Thymi from Δ*socs3* and WT mice were processed for histological analysis. In brief, tissues for immunofluorescence were embedded in OCT and frozen immediately in liquid nitrogen. Sections (8 μm) were fixed for 10 min in 4% PFA, washed in PBS and blocked in 2% BSA, 10% goat serum, 0.1% Tween-20 and 0.1% NaN_3_ in PBS.

Thymic sections were incubated with rabbit polyclonal antibodies to K5 and rat anti-K8 antibodies. Subsequently, sections were incubated with primary and fluorochrome-labeled secondary antibodies. After DAPI staining, sections were mounted with mounting gel (Invitrogen), and images of stained sections were captured using a Leica fluorescence microscope.

### Bone Marrow Radiation Chimeric Mice

Recipient WT and Δ*socs3* mice were irradiated 2x with 550 rad and received 5 × 10^6^ BM cell from either WT or Δ*socs3* mice. In some experiments CD45.1 congenic C57Bl/6 mice were used as bone marrow donors for irradiated WT or Δ*socs3* mice. Mice were kept for 3 weeks on antibiotics after transplantation (Tribrissen in drinking water). Tm was administered either 15 d before the BM transplantation or 15 d before sacrifice. Chimeric mice were sacrificed 70 days after the transplantation.

### Real Time-PCR

Transcripts were quantified by real time PCR as previously described (30). *Hprt* was used as a control gene to calculate the ΔC_t_ values for independent triplicate samples. The relative amounts of *socs3*/*hprt* transcripts was calculated using the 2^−(ΔΔCt)^ method. These values were then used to calculate the relative expression of cytokine mRNA Din treated or untreated cells and tissues.

### Statistical Analysis

Statistical analysis was performed using Graphpad Prism version 8. The *p-*values were calculated by two-tailed, unpaired Student's *t*-test or by one-way ANOVA analysis with a Kelch correction.

For the microarray analysis, a *p*-value cut-off of 0.05 and the Benjamini and Hochberg false discovery rate as multiple testing correction were performed to identify genes with statistically significant differences between WT and Δ*socs3* TECs. The Student-Newman-Keuls *post-hoc* test was used to identify the specific groups in which significant differential expression occurred. Genes that showed a change of 2-fold or greater were considered differentially expressed.

## Results

### SOCS3 Is Required for Maintenance of Thymus Structure and Thymocyte Differentiation

**Figure 1:**
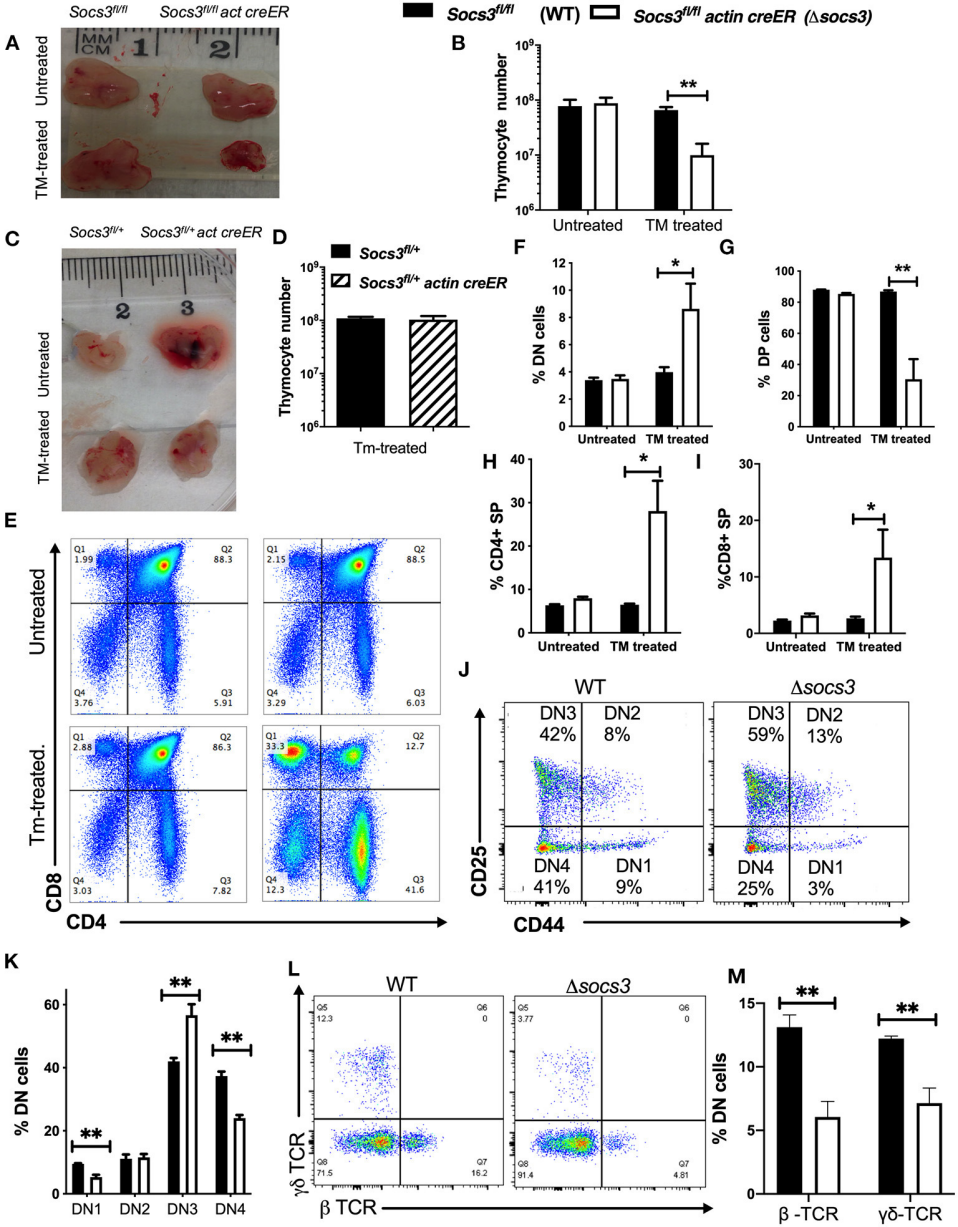
SOCS3 is required for maintenance of thymus structure and thymocyte differentiation. **(A)** Representative image and **(B)** mean total thymic cell numbers from Δ*socs3* and WT mice untreated or 7 days after the last dose of Tm. **(C)** Photograph from *Socs3*^*fl*/+^ and *Socs3*^*fl*/+^ *actin creER* thymi 7 days after Tm or left untreated. **(D)** Mean thymocyte numbers ± SEM from *Socs3*^*fl*/+^ and *Socs3*^*fl*/+^ *actin creER* mice 10 days after Tm treatment. Differences between groups are significant at *p ≤ 0.05, **p ≤ 0.01 ANOVA with Welch correction. **(E)** Representative dot plots and mean frequencies of lineage negative **(F)** DN, **(G)** DP, **(H)** CD4, and **(I)** CD8 SP thymocytes ± SEM in Δ*socs3* and WT mice (6 per group) treated or not with Tm. Four independent experiments were performed. These experiments were repeated more than 5 times with similar results. Differences between groups are significant at *p ≤ 0.05, **p ≤ 0.01 ANOVA with Welch correction. **(J)** Representative plot and **(K)** mean of frequency of DN1-DN4 Δ*socs3* and WT subpopulations ± SEM (*n* = 5 per group) defined by CD44 and CD25 expression. Differences between groups are significant at **p ≤ 0.01 unpaired Student's *t-*test. **(L)** Representative dot plots and **(M)** mean frequency ± SEM **(M)** of γδ and βTCR+ cells within the DN thymocyte population in Δ*socs3* and WT mice (*n* = 6 per group). Differences between groups are significant at **p ≤ 0.01 unpaired Student's *t-*test.

In order to study the role of SOCS3 in the production of T cells in the thymus, Δ*socs3* mice were treated with tamoxifen (Tm) for 5 days. Seven days after the last Tm dose mutant and *Socs3*^*fl*/*fl*^ (WT) thymi were compared. The cellularity of Tm-treated Δ*socs3* thymi was severely reduced. No reduction of thymic cellularity was observed in Tm-untreated Δ*socs3* control mice (Figures 1A,B). To exclude an off-target effect of Cre expression on thymocytes, *Socs3*^*fl*/+^ *actin creER* and *Socs3*^*fl*/+^ mice were generated. These animals showed similar thymic cellularity before and after Tm treatment (Figures 1C,D). Δ*socs3* mice showed no weight loss, spleen or lymph node enlargement, diahrrea and piloerection as compared to WT controls. Moreover, histopathological analysis of livers and lungs showed no signs of localized or disseminated inflammatory lesions (Supplementary Figure 1A).

Δ*socs3* mice showed an increased frequency of lineage negative DN cells among thymocytes (Figures 1E,F). Within the DN compartment, the frequency of Δ*socs3* DN3 cells was increased suggesting a block in thymocyte maturation at this stage of thymocyte development (Figures 1J,K). The frequency of γδ- and β-TCR expressing DN Δ*socs3* thymocytes was reduced compared to controls, which is line with this (Figures 1L,M).

The percentage of DP cells in Δ*socs3* thymi was dramatically reduced (Figures 1E,G), while the frequency of CD4 and CD8 SP cells were increased as compared to Tm-untreated Δ*socs3*, and to Tm-treated WT mice (Figures 1H,I). The numbers of all Δ*socs3* thymocyte populations (with exception of CD8 SP) and DN subpopulations were lower than controls (Supplementary Figures 1B–G).

Thus, SOCS3 plays a major role in thymocyte differentiation and in the maintenance of thymic cellularity.

### SOCS3 Expression by Thymic Stroma Cells Is Central for Thymus Maintenance and T Cell Differentiation

**Figure 2:**
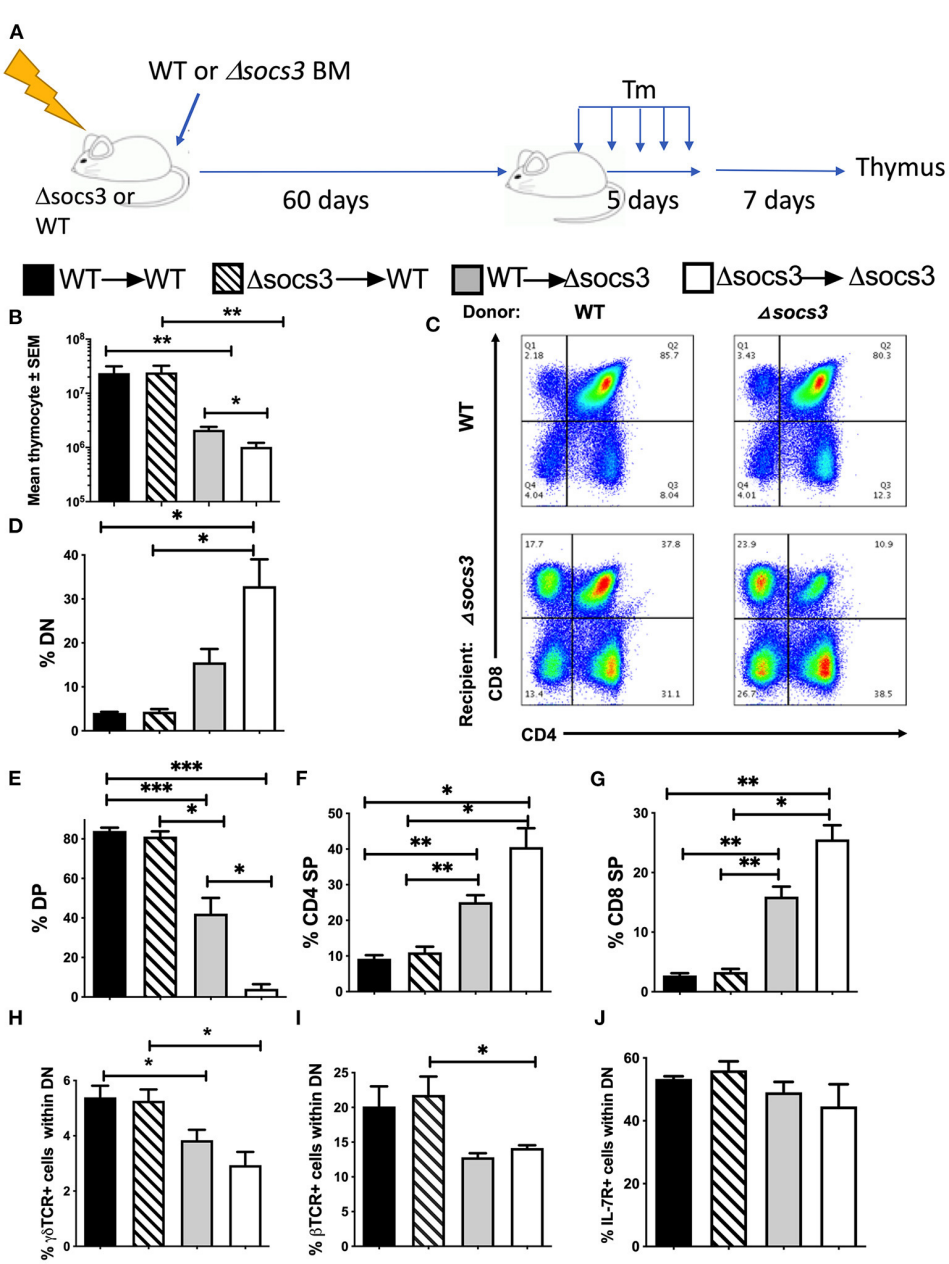
SOCS3 expression by non-hematopoietic cells is central for thymus maintenance and T cell differentiation. **(A)** Radiation bone marrow (BM) chimeras were generated using WT and Δ*socs3* mice as recipients or donors. Sixty days after transplantation, mice were treated with Tm and sacrificed 7 days after the last dose. **(B)** The mean thymic cell numbers ± SEM is depicted (*n* ≥ 5 per group). Differences between groups are significant at *p ≤ 0.05, **p ≤ 0.01 and ***p ≤ 0.01 Welch one way ANOVA. **(C)** Representative dot plots and **(D–G)** mean frequencies of DN, DP, CD4 SP, and CD8 SP thymocytes ± SEM in BM chimeric Δ*socs3* and WT mice are depicted (*n* ≥ 5 per group). Differences between groups are significant at *p ≤ 0.05, **p ≤ 0.01 Welch one-way ANOVA. **(H,I)** The mean frequency of **(H)** γδ and **(I)** β TCR+ cells within DN ± SEM BM chimeric mice is depicted. Differences between groups are significant at *p ≤ 0.05 Welch one-way ANOVA. **(J)** The mean % IL-7R+ cells within DN population ± SEM in BM chimeras of Δ*socs3* and WT is shown.

In order to determine whether SOCS3 expression in bone marrow (BM)-derived cells or non-hematopoietic cells is involved in the T cell differentiation in the thymus, BM radiation chimeras were generated and sacrificed 7 days after Tm-treatment completion (Figure 2A). The thymocyte numbers were reduced in Δ*socs3* recipient mice as compared to WT recipients. Instead, thymocyte levels in WT → WT and Δ*socs3* → WT were similar (Figure 2B), suggesting a role for SOCS3 in non-hematopoietic cells. In addition, Δ*socs3* → Δ*socs3* mice showed lower numbers of thymocytes as compared to WT → Δ*socs3* mice (Figure 2B).

The Δ*socs3* recipient mice contained lower percentages of DP, and higher of DN, CD4, and CD8 SP cells than WT recipients (Figures 2C–G). The percentages of DN, CD4, and CD8 SP were higher and DP cells were lower in Δ*socs3* → Δ*socs3* compared to WT → Δ*socs3* thymi suggesting a redundant role for hematopoietic SOCS3 expression in the control of thymic T cell development. The frequencies of thymocyte subpopulations in WT → WT and Δ*socs3* → WT mice were similar (Figures 2C–G). These results indicate that SOCS3 in non-hematopoietic cells is required to maintain T cell formation in the thymus. We observed lower numbers of DPs in Δ*socs3* than in WT recipients, while differences in other thymocyte populations were not statistically significant (Supplementary Figures 2A–D).

The percentage of γδ and αβTCR+ in DN thymocytes from Δ*socs3* recipient chimeric mice was reduced as compared to those of WT recipients (Figures 2H,I). Binding of IL-7 to its receptor (IL-7R) plays a non-redundant role in T cell development, by promoting the survival and proliferation of DN progenitors and of SPs cells during the positive selection (31). We observed that the frequency of IL-7R+ DN from Δ*socs3* or WT recipient mice was similar (Figure 2J). The frequencies of IL7R+ CD4 and CD8 SPs from Δ*socs3* recipients were higher than those of WT controls. The percentages of IL-7R+ CD4 and CD8 SPs from Δ*socs3* → Δ*socs3* were higher than those from WT → Δ*socs3* mice, while that of IL-7R+ CD4 and CD8 SP from Δ*socs3* → WT and WT → WT mice were similar, suggesting that SOCS3 in non-hematopoietic cells regulates IL-7R expression during late stages of thymocyte development (Supplementary Figures 2E–G).

A sequence of events within the SP stage is necessary before T cell export occurs: Maturation of SP thymocytes is associated with downregulation of CD24 and upregulation of Qa-2 molecules (3). CD24^low^Qa2^high^ SPs thymocytes proliferate when triggered through the TCR prior to export to the periphery (32). CD4 and CD8 SPs from Δ*socs3* thymi contained higher Qa2 (Supplementary Figures 3A–D) and lower CD24 levels (Supplementary Figures 3E–H) than controls. Thus, Δ*socs3* SP cells display a higher maturation level than WT controls.

T regs cells that develop in, and emerge from, the thymus maintain self-tolerance and prevention of autoimmune disorders. We found that the frequency of FOXP3+ cells within Δ*socs3* and WT CD4 SPs was similar, suggesting that SOCS3 does not preferentially regulate the development of Tregs in the thymus (Supplementary Figure 3I).

**Figure 3:**
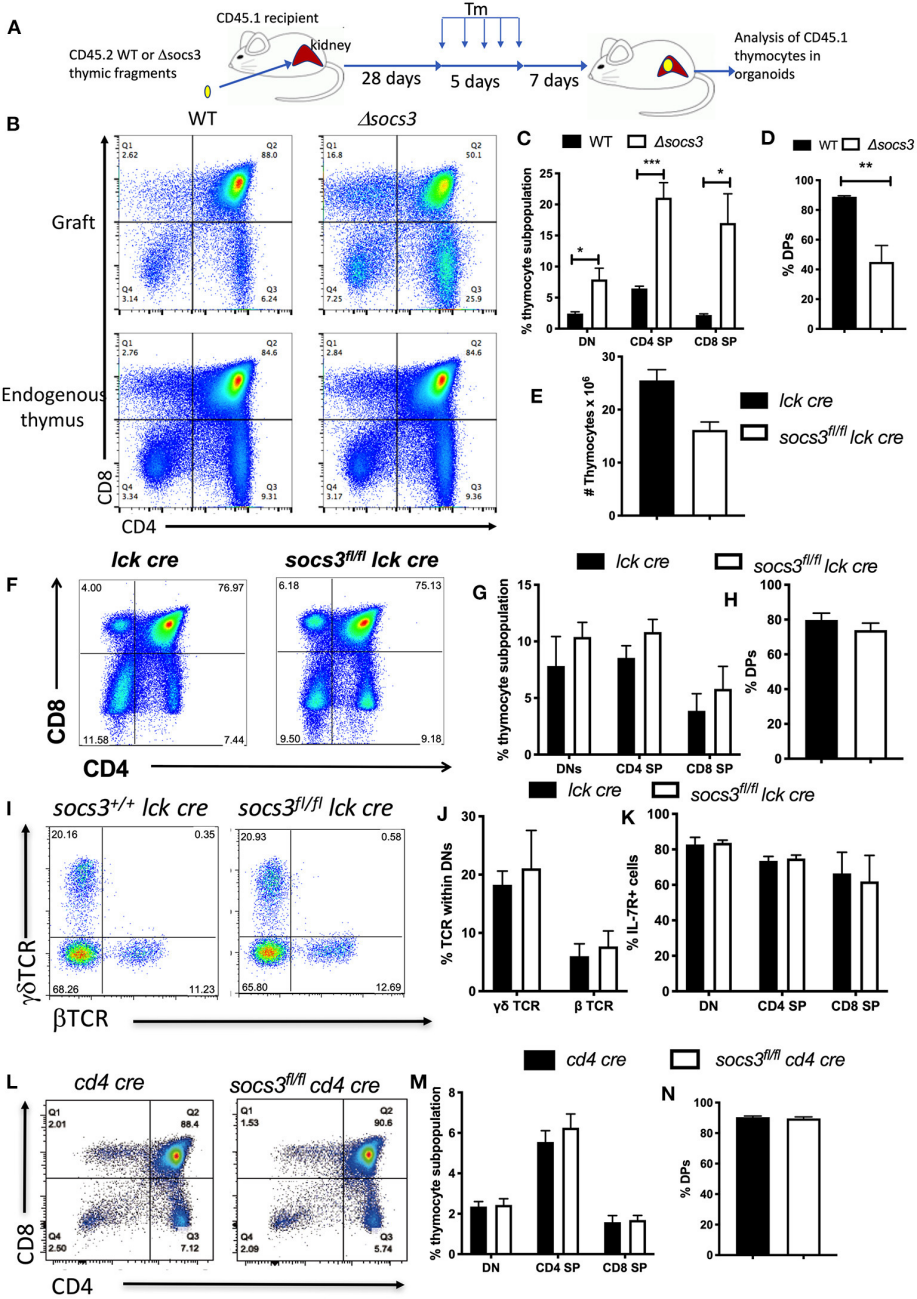
SOCS3 expression in thymic stroma but not in thymocytes is required for T cell maturation in the thymus. **(A)** Thymic fragments from 1 to 3 days old WT and Δ*socs3* mice were transplanted into the kidney capsule of CD45.1+ mice. Four weeks after the implantation mice were treated with Tm daily for 5 days. Seven days after the last Tm dose mice were sacrificed and the thymic organoid grafts and the “endogenous” thymus were explanted for analysis. **(B)** Representative dot plots shown compare the thymocytes from mice grafted with CD45.2 WT and Δ*socs3* thymic fragments and the endogenous thymus (gated on CD45.1+ recipient cells), 7 days after the last Tm dose. **(C,D)** The mean frequencies of CD45.1+ DN, DP, CD4 SP, and CD8 SP ± SEM in the thymic grafts obtained as indicated above are shown. Differences between groups are significant at *p ≤ 0.05, **p ≤ 0.01, ***p ≤ 0.001 (unpaired Student's *t-*test). These experiments were performed 3 times. **(E)** The mean number of *Socs3*^*fl*/*fl*^ *lck cre* and *Socs3*^+/+^ *lck cre* thymocytes ± SEM (at least 6 per group) are depicted. **(F)** Representative dot plots and **(G,H)** the mean frequencies of *Socs3*^*fl*/*fl*^ *lck cre* and *lck cre* DN, DP, CD4 SP, and CD8 SP ± SEM are shown. **(I)** Representative dot plots and **(J)** mean frequencies ± SEM of *Socs3*^*fl*/^ *lck cre* and *Socs3*^+/+^*lck cre* γδ and β TCR DN cells ± SEM are depicted. **(K)** The frequency of IL-7R+ *Socs3*^*fl*/*fl*^ *lck cre* and *Socs3*^+/+^ *lck cre* thymocyte populations ± SEM mice are shown. **(L)** Representative dot plots and **(M,N)** the mean frequencies of DN, DP, CD4, and CD8 SP thymocytes ± SEM in *Socs3fl/fl cd4 cre* and *Socs3*^+/+^ *cd4 cre* mice are shown.

Next, we investigated whether the expression SOCS3 in thymic stromal cells regulates thymic T cell development. For this purpose, WT or Δ*socs3* CD45.2 thymic fragments were implanted under the kidney capsule of CD45.1 mice. Four weeks after implantation mice were treated with Tm for 5 days. The engraftment was resected 7 days after Tm treatment, and the presence of CD45.1+ thymocytes in the kidney organoid evaluated (Figure 3A, Supplementary Figures 4A,B). The frequencies of CD45.1+ thymocyte DN, DP, SP, and subpopulations in the WT graft were similar to that of the endogenous thymus of recipient mice, indicating a functional T cell development in the ectopic thymus (Supplementary Figures 4C,D), in agreement with previous studies (33). The Δ*socs3* graft instead showed a diminished frequency of DP and increased DN and CD4+ and CD8+ SP levels as compared with those in the WT graft or the endogenous thymus (Figures 3B–D).

To confirm that SOCS3 in thymocytes plays a minor role if any in thymic T cell development, thymocyte populations in *Socs3*^*fl*/*fl*^ *lck cre* and *Socs3*^*fl*/*fl*^ *cd4 cre* mice were analyzed. Lck is expressed by the early DN (34) while CD4 will be first expressed at the late DN to DP stage of thymocyte development. *Socs3*^+/+^*lck cre* (*lck cre*) were used as controls for *Socs3*^*fl*/*fl*^*lck cre* thymi since lck cre expression alters thymic T cell development (35). The numbers of thymocytes in *Socs3*^*fl*/*fl*^*lck cre* and *lck cre* mice were similar (Figure 3E). The frequency of thymocyte DN, DP, and SP subpopulations, the levels of IL-7R in DN and the frequency of β- and γδ TCR+ cells in *Socs3*^*fl*/*fl*^ *lck cre* and control DN were similar (Figures 3F–K). In agreement with these results, the frequencies of thymocyte DN, DP and SP subpopulations in *Socs3*^*fl*/*fl*^ *cd4 cre* and *cd4 cre* animals were also similar (Figures 3L–N). The percentages of βTCR+ and γδTCR+ *Socs3*^*fl*/*fl*^ *cd4 cre* and *Socs3*^+/+^*cd4 cre* DN thymocytes were also comparable (Supplementary Figures 4E,F). Altogether, SOCS3 in thymic stroma is critical for T cell formation in thymus.

### Decreased Thymocyte Proliferation, Differentiation, and Increased Frequency of Apoptotic Thymocytes in Δsocs3 Mice

**Figure 4:**
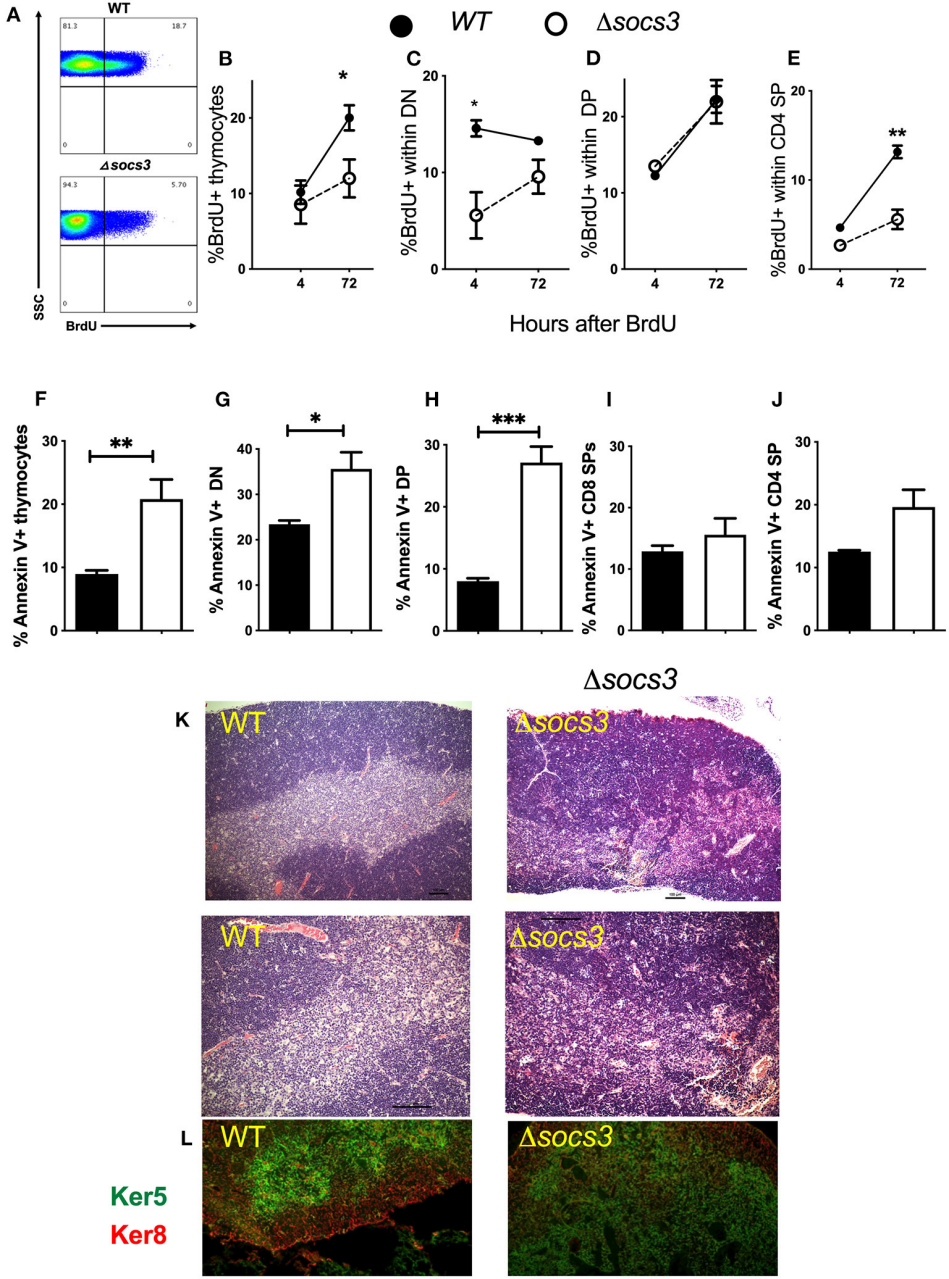
Decreased proliferation, differentiation and increased frequency of apoptosis in Δsocs3 thymocytes. **(A)** A representative histogram shows BrdU+ thymocytes from Δ*socs3* and WT mice measured 72 h after BrdU administration. **(B)** The mean frequency of BrdU+ thymocytes ± SEM from WT and Δ*socs3* mice (*n* = 5 animals per groups) 4 and 72 h after BrdU inoculation are depicted. The mean frequency ± SEM of BrdU+ cells within **(C)** DN, **(D)** DP, and **(E)** CD4 SP Δ*socs3* and WT thymocytes at 4 and 72 h after BrdU administration is shown. The mean frequencies of Annexin V+ **(F)** total thymocytes and **(G)** DN, **(H)** DP, **(I)** CD4 SP, and **(J)** CD8 SP subpopulations ± SEM in Δ*socs3* and WT mice (at least 5 per group) are shown. Differences between WT and Δ*socs3* thymocytes are significant at *p ≤ 0.05, **p ≤ 0.01, ***p ≤ 0.001, two-way ANOVA and unpaired Student's *t-*test. **(K)** The haematoxylin and eosin staining depicting the organization of thymi from WT as compared to Δ*socs3* mice. A representative micrograph from tissues from 5 mice per group analyzed is shown. Scale bar: 100 μm. **(L)** Double immunolabelling of keratin 5 and keratin 8 targeting the thymic cortex and medulla, respectively, in WT and Δ*socs3* mice is shown.

The proliferation and differentiation of Δ*socs3* and WT thymocytes was investigated after a single BrdU pulse performed 7 days after Tm administration. The frequency of BrdU+ WT thymocytes was increased from 4 to 72 h after the administration of the nucleoside showing proliferation of labeled cells (Figure 4B). The frequency of BrdU+ thymocytes at 72 h after administration was lower in Δ*socs3* than in WT controls (Figures 4A,B).

We then analyzed BrdU incorporation within the different thymocyte subpopulations (Supplementary Figure 3G). The frequency of BrdU+ DN measured 4 h after the pulse was lower in Δ*socs3* than in control thymocytes (Figure 4C), suggesting a lower DNA synthesis in the absence of SOCS3. A tendency toward a reduction of the proportion of BrdU+ DN was observed when comparing 4–72 h time points after BrdU pulse in WT thymocytes, while the opposite was observed for Δ*socs3* thymocytes (Figure 4C). This further supports a block in the Δ*socs3* thymocyte differentiation at the DN stage. The frequencies of BrdU+ DP Δ*socs3* and WT thymocytes increased from 4 to 72 h after BrdU administration (Figure 4D). The percentage of BrdU+ CD4+SPs augmented from 4 to 72 h after the nucleoside administration (Figure 4E). The fraction of BrdU+ Δ*socs3* CD4 SPs at 72 h after administration was lower than that of WT controls (Figure 4E).

To study whether the thymus hypoplasia in Δ*socs3* mice was due to increased cell death, the proportion of Annexin-V+ thymocytes was measured. We found higher frequencies of Annexin-V+ cells in the Δ*socs3* thymocytes as compared to WT controls (Figure 4F). The percentage of Annexin-V+ DN and particularly that of DP from Δ*socs3* thymi was increased as compared to WT controls (Figures 4G,H). On the other hand, the Annexin V labeling in CD4 and CD8 SP thymocytes from Δ*socs3* and WT mice was similar (Figures 4I,J). Thus, SOCS3 deficiency in the thymus results in a reduced proliferation, blocked differentiation and increased apoptosis of the thymocyte populations.

### Abnormal Thymus Architecture in Δsocs3 Mice

Since the defect of thymocyte maturation in Δ*socs3* mice was assigned to the thymic stroma, the levels of cTECs and mTECs in Δ*socs3* thymi were then compared. We found that the frequencies of TECs (defined as CD45-EpCAM+ cells) were higher in Δ*socs3* than in WT thymi (Supplementary Figures 5A,B). The total numbers of TECs in Δ*socs3* and control thymi were similar, and the increased TEC frequencies in Δ*socs3* thymi are explained by the diminished Δ*socs3* thymocytes numbers (Supplementary Figure 5C).

Δ*socs3* and WT thymi showed similar numbers and frequencies of cTECs and mTECs (distinguished by UEA-1 binding and Ly51 expression) (Supplementary Figures 5D,E). cTECs with high levels of MHCII and CD40 and mTECs with high CD80 and MHCII levels are considered functionally mature (1). The MHC-II expression level was lower in Δ*socs3* mTECs compared to WT controls, while similar MHCII levels were measured in mutant and WT cTECs (Supplementary Figures 5F–H).

Hematoxylin and eosin (H&E)-stained sections revealed densely packed cells in the Δ*socs3* thymi. Whereas, cortex and medulla are distinguishable in WT thymi, the cortico-medullary junction in Δ*socs3* thymi is imprecise (Figure 4K). Keratin-5 (K5) and keratin-8 (K8) are specific markers for mTECs and cTECs, respectively. IHC staining with K5 and K8 revealed that the cTEC and mTEC subsets were present in Δ*socs3* thymi, however the fraction of area with an overlapping staining of K5 and K8 were more extensive in Δ*socs3* compared to WT mice (Figure 4L).

### Stromal SOCS3 Regulates Numbers and Frequencies of Peripheral Naive T Cell Subpopulations and Their Activation

**Figure 5:**
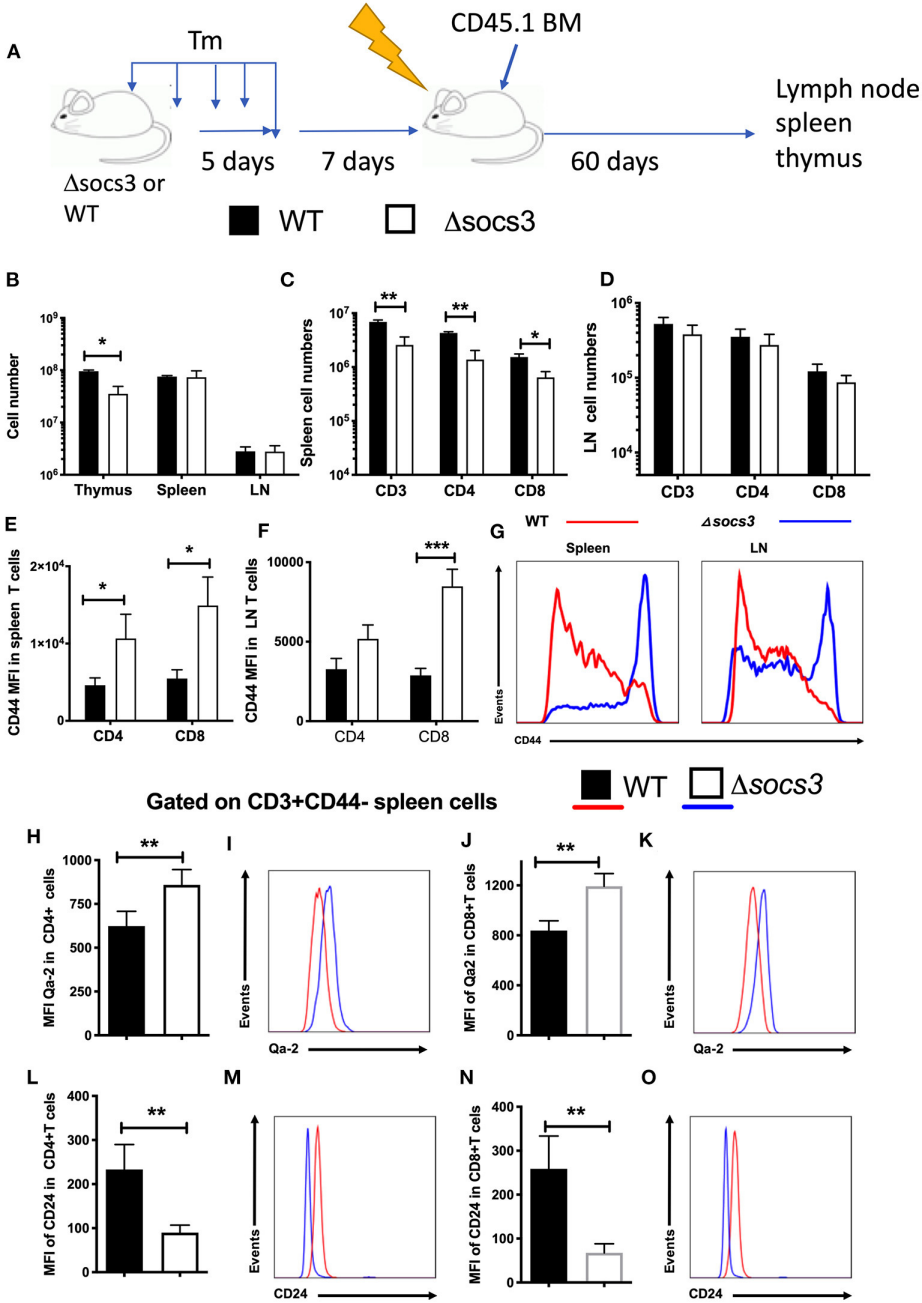
Non-hematopoietic SOCS3 expression regulates the frequency recent thymic emigrants and of naïve peripheral T cells. **(A)** WT or Δ*socs3* mice treated with Tm and 7 days after the last dose of Tm were irradiated and transplanted with CD45.1+ WT bone marrow cells. Mice were sacrificed 60 days after BM transplantation. **(B)** The mean number of thymus, spleen and lymph node cells ± SEM of transplanted mice are depicted (*n* = 5 per group). The mean number ± SEM of CD45.1+ and CD3 gated CD4, CD8, and γδ T cells in **(C)** spleens and **(D)** lymph nodes of radiation chimeric mice treated as described above is shown (*n* = 5 per group). The mean MFI of CD44 in CD45.1 gated CD4 or CD8 T cells in spleens **(E)** and lymph nodes **(F)** is shown. **(G)** A representative histogram of CD44 in CD8 T cells in lymph node or spleen cells from Δ*socs3* and WT mice. The mean MFI and representative histograms of Qa-2 **(H–K)** and CD24 **(L–O)** expression in naïve CD4 **(H,I,L,M)** and CD8 **(J,K,N,O)** splenic T cells of WT and Δ*socs3* mice 7 days after Tm treatment are depicted. Differences between groups are significant at *p ≤ 0.05, **p ≤ 0.01, ***p ≤ 0.001, unpaired Student's *t*-test.

Then, whether SOCS3 deletion in stromal cells regulated the levels of naïve T cells in the secondary lymphoid organs was further investigated. For this purpose, Δ*socs3* or WT CD45.2 mice were first treated with Tm. Ten days after Tm treatment, mice were irradiated and transplanted with CD45.1+ BM cells (Figure 5A). The levels of CD45.1+ T cells in the lymph nodes and spleens were studied 60 days after transplantation. The thymus of transplanted Δ*socs3* mice showed a reduced cellularity as compared to that of WT controls (Figure 5B), albeit differences were lower compared to those analyzed 10 days after Tm administration. The thymocyte subpopulations numbers in Δ*socs3* and WT chimeric recipients was similar (Supplementary Figure 6A). The spleen and lymph node cell numbers in transplanted Δ*socs3* and WT mice were similar (Figure 5B). The levels of CD45.1+ CD4 and CD8 T cells in spleens (but not in lymph nodes) of Δ*socs3* recipient mice were diminished as compared to WT controls (Figures 5C,D). In spleens and lymph nodes of Δ*socs3* recipient mice, the expression of CD44 in CD4 and CD8 T cells was enhanced compared to controls (Figures 5E–G). This indicates that lymphoid organs of BM-transplanted Δ*socs3* mice contain lower frequencies of naïve T cells compared to those of WT controls when analyzed 70 days after Tm treatment.

T cell differentiation of naïve T cells continues post-thymically, with progressive maturation of both surface phenotype and immune functions. Recent thymic emigrants lose CD24 and gain of Qa2 and CD45RB expression upon transition to mature naïve T cells (36, 37). CD4 and CD8 SP thymocytes expressed lower levels of Qa-2 (Supplementary Figures 6B,C) and higher levels of CD24 (Supplementary Figures 6D,E) than spleen or LN CD44 neg T cells, in coincidence with previous results (36). The expression level of Qa-2 was higher in naïve CD4+ or CD8+ spleen cells from Δ*socs3* mice 7 and 14 days after Tm treatment compared to those in WT spleens (Figures 5H–K). Instead, naïve spleen T cells from Δ*socs3* mice showed lower levels of CD24 (Figures 5L–O). Thus both, the frequency of peripheral naïve T cells and the levels of recent thymic emigrants within this population are regulated by SOCS3 expression.

### SOCS3 in Thymic Epithelial Cells Binds to the E3-Ubiquitin Ligase TRIM21

**Figure 6:**
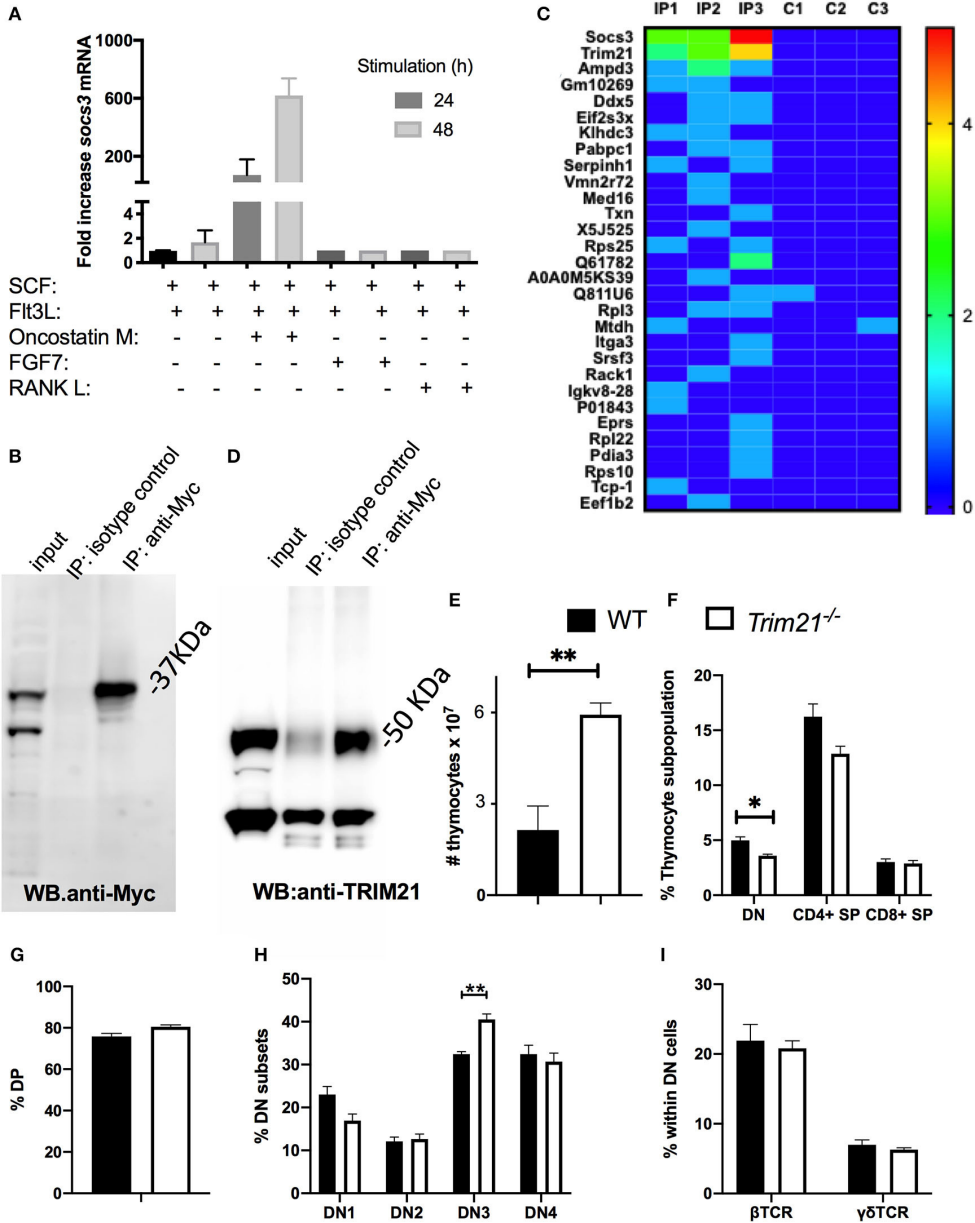
SOCS3 in thymic epithelial cells binds to TRIM21. **(A)** OP9-DL1 cells were incubated with either SCF, Flt3L, OMS, FGF7, or RANK. Twenty four and forty eight hour after stimulation, the total mRNA was extracted and *Socs3* and *Hprt* mRNA levels measured by real time RT-PCR. The mean relative fold induction of *Socs3*/ *Hprt* mRNA ratio in stimulated and unstimulated cells are depicted. **(B)** OP9-DL1 cells were transfected with an Myc-SOCS3 plasmid. The western blot of transfected cell lysates before and after immunoprecipitation with anti-myc or an isotype controls is shown. **(C)** Heat map of ranking proteins identified after tandem mass spectrometry in immunoprecipitates from *socs3-*transfected OP9-DL1 cells with anti-myc or isotype controls. Three replicates per condition were analyzed. Ranking was calculated with an algorithm weighing the number of replicates in which the protein was detected in the IP and in the control group, the number of peptide spectral matching the protein, the number of unique peptides and the intensity (the area under the curve). **(D)** Anti-SOCS3 or anti-isotype immunoprecipitates were analyzed by immunoblot using anti-TRIM21. **(E)** The mean number of WT and *Trim21*^−/−^ thymocytes ± SEM are depicted (*n* ≥ 5 per group). Differences between groups are significative (**p ≤ 0.01 unpaired Student's *t*-test). **(F,G)** The mean frequency of thymic DN, DP, and SP subpopulations ± SEM in *Trim21*^−/−^and WT thymi (*n* = 5 per group). **(H)** The mean of frequency ± SEM of DN1-DN4 subpopulations (defined by CD44 and CD25 expression) in *trim21*^−/−^and WT thymi (n≥5 per group). **(I)** The mean % of β and γδ TCR+ cells within the DN thymocytes of WT and *trim21*^−/−^ mice (*n* ≥ 5 per group). Differences between groups are significant at **p ≤ 0.01 unpaired Student's *t*-test.

We next studied the binding partners of SOCS3 in TECs. We observed that OP9 epithelial cells expressing the Notch ligand-Delta like 1 (OP9-DL1) cultured in conditions that enable thymopoiesis (with SCF and Flt3l) (38) express high levels of SOCS3 when OSM, (but not RANK-L or FGF7) was added into the culture (Figure 6A) and therefore choose these cells for our investigation.

OP9-DL1 cells were transfected with SOCS3-Myc expressing plasmids. The expression of SOCS3-Myc was immunoprecipitated (IP) from lysates of transfected cells (Figure 6B) and the IP proteins were tryptically digested and analyzed by liquid chromatography-mass spectrometry. Proteins were ranked with regards to their presence in replicates and the spectral counting. Three proteins were found in all 3 replicate target IP's of transfected cells and 6 proteins were identified in 2 out of 3 of the target IP's but not in the negative control.

One of the 3 proteins present in all replicates was SOCS3, and other was TRIM21 (Figure 6C). SOCS proteins act as substrate adapters for ubiquitination and proteosomal degradation of different receptors (11). Tripartite motif (TRIM) proteins, including TRIM21, have been implicated in multiple cellular functions that rely on their E3-ubiquitin ligase activity (39). Other members of the TRIM family have been shown to interact with SOCS proteins (40, 41). We validated the presence of TRIM21 in the SOCS3 IP using anti-TRIM21 antibodies in the WB (Figure 6D). Then, whether TRIM21 could play a role in T cell formation in the thymus was studied. Contrary to Δ*socs3*, thymi from *trim21*^−/−^ mice showed an increased number of thymocytes (Figure 6E) and reduced frequency of DN cells (Figure 6F), while the frequencies of other thymocytes populations in WT and *Trim21*^−/−^ were similar (Figures 6F–H). The frequency of γδ and β TCR+ DN cells in a WT and *Trim21*^−/−^ mice was also similar (Figure 6I).

### Reduced Central T Cell Tolerance Transcript Levels and Increased Expression of IL-6 Cytokine Family Regulated Genes in Δsocs3 TECs

**Figure 7:**
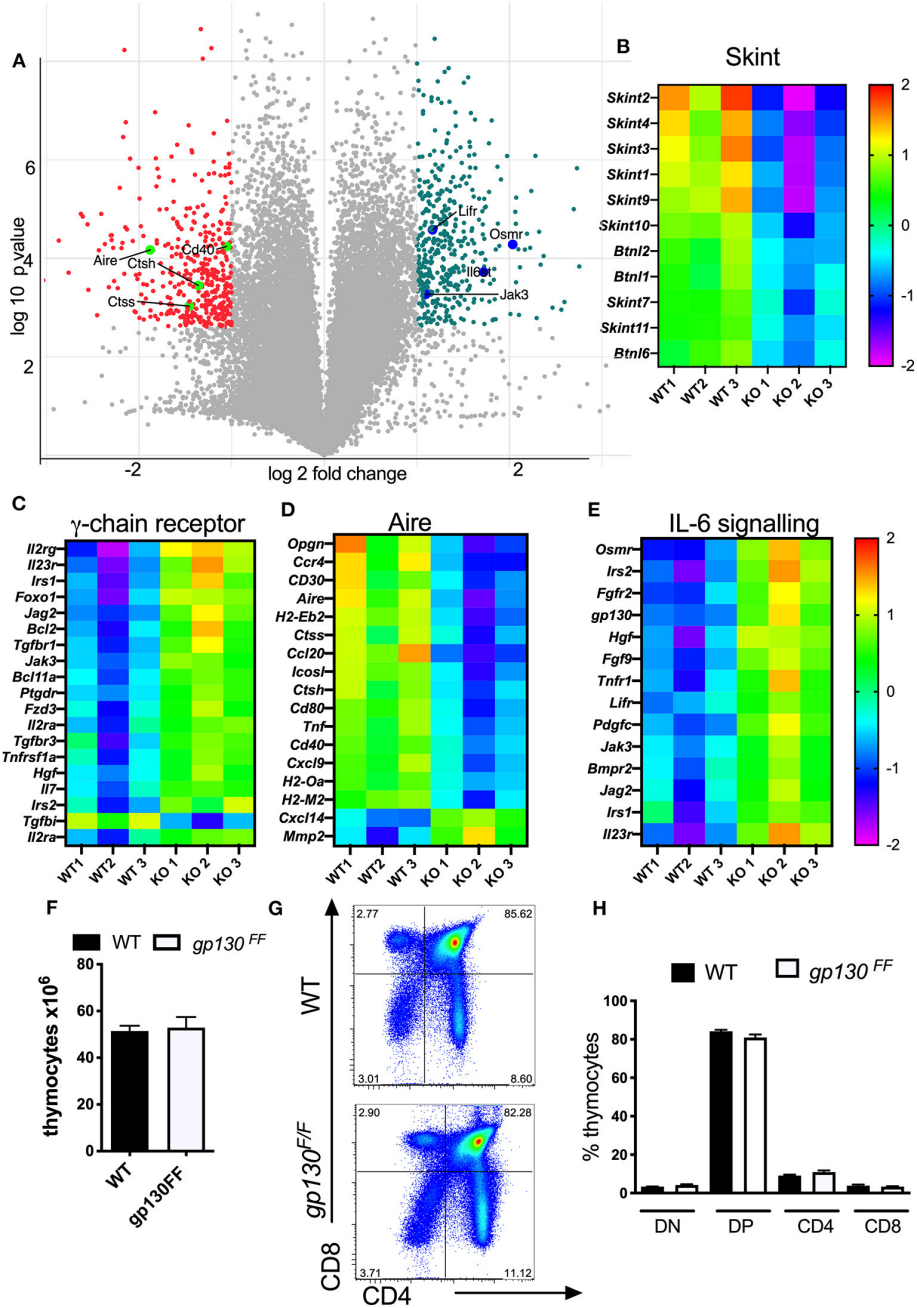
Δsocs3 TECs show diminished expression of genes involved in T-cell selection and increased levels of those from the IL-6 family. **(A)** Volcano plot of the gene expression comparing the log_2_ fold change (FC) differences and the statistical significance (log_10_ probability) of transcript levels from three independent Δ*socs3* and WT TECs samples. Highly dysregulated in absence of SOCS3 are labeled in pink. The Student-Newman-Keuls *post-hoc* test was used to identify the specific groups in which significant differential expression occurred. The heat maps of **(B)** skint, **(C)** common γ-chain receptor **(D)** aire, and **(E)** IL-6 signaling pathways representing transcript levels in mutant and control TECs are depicted. Data were normalized for each gene by mean subtraction of log_2_ transformed values. Gene names are shown adjacent to heat maps. For all genes included in the heat maps, differences between WT and Δ*socs3* TECS are statistically significant. **(F)** The mean thymocyte number, **(G)** a representative dot plot and **(H)** the frequencies of DN, DP, CD4, and CD8 SP thymocytes ± SEM in *Gp130*^*F*/*F*^ and WT mice (*n* = 5 per group).

The genome wide transcriptome of Δ*socs3* and WT CD45-EpCAM+ TECs was then compared. Enriched TEC populations were negatively selected with CD45 magnetic beads and subsequently sorted as EpCAM+ cells. The whole genome transcriptome of three independent samples per group was determined. Among 9,392 transcripts expressed above threshold levels in both groups, 703 were ≥ 2-fold and significantly differently (*p* < 0.05) expressed, 367 higher and 336 lower in WT than in Δ*socs3* WT TECs (Figure 7A). A GO analysis indicated several families with different transcript levels (Supplementary Table 1). Expression levels of 8 out of 11 transcripts of the Skint- and 3/ 6 of the related butyrophylin-like gene family were increased in WT TECs (Figure 7B). Genes within cytokine receptor interaction pathways were increased while others from the same GO were reduced in WT TECs (Supplementary Tables 2A–D). The relative levels of several genes stimulated via the common cytokine receptor γ-chain (IL-2R_γ*c*_) signaling pathway were decreased in WT compared to Δ*socs3* TECs (Figure 7C).

The expression level of genes involved in T cell selection (such as *Aire, CD40, CD80*); several genes from the MHC-II locus and *Cathepsin m* and -*l* (involved in antigen presentation); chemokines and chemokine receptors as well as members of the TNF-receptor family involved in thymocyte development were all higher in WT than in Δ*socs3* TECs (Figure 7D).

The SOCS3 regulation of STAT3 activation by the gp130 receptor signaling controls inflammatory responses via the IL-6 receptor family (42). In line with this, several genes related to IL-6 signaling or regulated by SOCS3 such as *Gp130, Osmr, Lifr, Irs1, Irs2*, and *Lifr* were reduced in WT TECs (Figure 7E). *Socs3* mRNA levels were significantly reduced in Δ*socs3* TECs as compared to controls. We then asked if SOCS3 binding to gp130 was critical for T cell generation in the thymus. Cells from *Gp130*^*F*/*F*^ mice, harboring a mutation that ablates SOCS3 binding to gp130, show exaggerated STAT3 responses (25). We found that the thymus cellularity, the frequency and numbers of different thymocyte subpopulations in *Gp130*^*F*/*F*^ and WT controls were similar (Figures 7F–H). Thus, SOCS3 control of gp130 signaling is redundant for the differentiation of T cells in the thymus.

## Discussion

We here show that SOCS3 is critical in the regulation of T cell formation in the thymus and for maintenance of thymus architecture by using mice with a Tm-inducible *Socs3* gene deletion. Off-targets effects of cre and Tm treatment previously reported by others and us were carefully ruled out (35, 43, 44).

The thymus is highly sensitive to acute stress, and malnutrition, pregnancy, infection, autoimmune diseases and cancer might result in the reduction in thymus size (45). Our data demonstrate an important role for SOCS3 in thymus homeostasis under physiological conditions.

Mice lacking SOCS3 in the skin developed exacerbated chronic inflammation (46, 47). Δ*socs3* mice showed neither clinical signs nor inflammatory histopathology in other organs. This might be explained by the fact that we recorded thymic changes at an early time point, 7–10 days, after the induction of *Socs3* gene deletion. In comparison, mice lacking SOCS3 in hematopoietic cells or in keratin-5-expressing epithelial cells that showed inflammatory disease at 3 or 4 months of age (14, 46). In line with our observation, mice deficient in SOCS3 in neurons (48), glia (48), endothelial cells (49), smooth muscle cells (50), gut epithelial cells (51), myeloid cells (21) or B cells (52) showed no spontaneous autoimmune or inflammatory disease.

Δ*socs3* mice displayed a striking reduction in the numbers of all thymocyte subpopulations. The reduced proliferation of Δ*socs3* DN and the increased frequency of DN3 cells indicate that SOCS3 regulates early stages of T cell development in the thymus. This is further evidenced by very low frequency and the dramatically increased level of apoptosis of remaining Δ*socs3* DP cells. The BrdU labeling also revealed the impaired differentiation to of thymocytes.

The lower frequencies of naïve CD44^low^ T cells in secondary lymphoid organs of Δ*socs3* mice suggest a lymphopenia-triggered homeostatic proliferation of naïve T cells that may acquire a memory phenotype even in the absence of antigenic stimulus (53). The reduced CD24 and increased Qa-2 levels in Δ*socs3* SPs and in peripheral CD44 negative T cells suggest the accumulation of pre-recent thymic emigrants (RTE) in the thymus and RTE in secondary lymphoid organs. This might compensate for a dysfunctional T cell formation in the Δ*socs3* thymus: pre-RTEs display a selective survival advantage over other thymocyte populations and both pre-RTEs and RTEs are essential for the establishment and maintenance of a self-tolerant and a diverse and functional T cell repertoire (54, 55).

The thymic involution was recapitulated in BM chimeric mice in which recipient cells were SOCS3-deficient. Moreover, thymus transplantation experiments showed that SOCS3 in thymic stroma cells is required during T cell formation, while the role of SOCS3 in thymocytes or in hematopoitic cells in T cell development was minor and redundant and it could only be observed in chimeric mouse when Δ*socs3* mice were used as recipients.

Whereas, the numbers of cTECs and mTECs in the mutant thymus remained unaltered, the thymus structure was altered in Δ*socs3* thymi. A clear demarcation of medulla and cortex was absent, and an aberrant co-localization of cTECs and mTECs was observed in Δ*socs3* thymi.

Here we show that SOCS3 binds to TRIM21 in OP9-DL-1 epithelial cells. SOCS and TRIM proteins target the receptor complex for ubiquitination and proteasome- degradation, acting as substrate adaptors (56). SOCS3 in TECs could promote TRIM21 degradation or TRIM21 might target SOCS3 for degradation thus impairing the JAK kinase inhibition. The increased numbers of thymocytes and reduced DN population frequencies are dissimilar to the features of Δ*socs3* thymi and suggest that TRIM21 might reduce SOCS3 stability in TECs, a possibility that remains to be confirmed. Another member of the family, TRIM8 has been shown to interact with SOCS1 decreasing its stability and levels (40). TRIM8 interacted also with PIAS3 and Hsp90β regulating STAT3 activation (57, 58). TRIM21 is expressed in the thymus (26), and the alterations in frequencies and numbers of thymocyte subpopulations can reflect an altered T cell development in the thymus, which might contribute to the autoimmune phenotype of *Trim21*^−/−^ mice (26).

SOCS3 binds to gp130 hampering the response to cytokines of the IL-6 family (10). Transgenic animals overexpressing IL-6 cytokine family members LIF, IL-6 or OSM show thymus involution, and similar results were observed after administration of recombinant cytokines (59, 60). These cytokines present in the thymic microenvironment, are produced by TECs, increase with age and have been associated to thymic atrophy (60). Gp130 is expressed ubiquitously on thymocytes (61) and on thymic epithelium (62) and thymic atrophy caused by these cytokines was reverted by gp130 neutralization. On the other hand, gp130 was shown to be required for proper thymic formation (63), and deficiency of OSM or IL-6 resulted in thymic hypoplasia, altered medullary structure and autoreactivity (64–66). Thus, the IL-6R family of cytokines in physiological states protects the thymic structure but might suppress thymic functions at high concentrations. In our hands, *Gp130*^*F*/*F*^ mice showed no differences in thymocyte populations numbers and frequencies, indicating that SOCS3 signaling through gp130 is redundant in SOCS3-mediated thymus maintenance. This is in agreement with previous data showing that thymi from *Gp130*^*F*/*F*^ and WT controls have similar cellularity (47). Yet, *Lifr* and *Osmr* transcripts were increased in Δ*socs3* TECs indicating that a more indirect role of SOCS3 increasing these or transcripts for *Irs1, Irs2, Tgfr1, Hgf* and *Igf1* all previously suggested to be involved in thymic formation or maintenance.

Skint and butyrophilin are members of the butyrophilin-like subfamily of B7-related proteins that, similar to MHC or CD1, modulate T cell functions and are considered to be co-stimulatory molecules (67). Butyrophilins-like molecules are expressed by TECs and regulate thymic T cell selection, particularly that of γδ T cells (68, 69). We observed a remarkable reduction in the levels of several transcripts of the butyrophilin family in Δ*socs3* TECs. This suggests that SOCS3 in TECs control T cell maturation in the thymus by regulating the levels of butyrophilin-like proteins.

The alterations in Δ*socs3* thymi resemble the age-associated changes in thymopoiesis, in which defects within the thymic stromal niche result in impaired T cell development. Several studies have demonstrated that with age, the thymic microenvironment undergoes structural, phenotypical, and architectural changes (70), including the down regulation of MHC-II (71), which we observed in Δ*socs3* mTECs. While our data indicate that SOCS3 affects thymocyte maturation already at DN stage, a role of the molecule at later stages of thymic maturation cannot be ruled out. The autoimmune regulator AIRE transcription factor, the cell surface receptors CD40 and RANK mediate mTECs development and central tolerance, whereas LT-β receptor is required for mTEC differentiation and expression of adhesion molecules needed for proper localization of T cell precursors. AIRE+ mTEC^hi^ subsets are further subdivided based on osteoprotegerin (OPG) expression. OPG regulates the cellularity of mTECs and the size of the medullary region in the thymus, by attenuating the RANK-mediated mTECs proliferation (7). In line with this, the levels of *Aire*, the members of the TNFR superfamily *Opg, Cd40, Rank*, and *CD30*, chemokines like *Ccl20* and transcripts coding for MHC molecules controlling T cell selection in the thymus were all diminished in Δ*socs3* TECs (72).

In summary, SOCS3 expression by the thymic stroma, but not in thymocytes is required for the maintenance of the thymic architecture and the correct localization and maturation of cTECs and mTECs. SOCS3 inhibits the expression of a number of genes in TECS (including *Irs1, Irs2, Il23r*, or *Lepr*) that regulate thymocyte differentiation and promotes several genes involved in central tolerance. In absence of SOCS3 in the thymic stroma, thymocyte proliferation and differentiation are hampered. Δ*Socs3* thymocytes accumulate at the DN stage where generation of both β- and γδ TCR DN cell frequencies are reduced. The diminished frequency and survival of DP cells may lead to a deficient differentiation of SPs cells observed. Consequently, the production of recent thymic emigrants and the frequency of naïve T cells are reduced.

Altogether, we here show that SOCS3 plays a central role in maintaining the maturation and morphology and tissue distribution of TECs. Our results indicate that SOCS3 through this role provides niches for thymocyte maturation, and consequently is required for proper thymocyte development and naïve T cell export.

## Data Availability Statement

The datasets presented in this study can be found in online repositories. The names of the repository/repositories and accession number(s) can be found at: https://www.ncbi.nlm.nih.gov/geo/, GSE165216.

## Ethics Statement

The animal study was reviewed and approved by Stockholms North Region Animal Research Ethic Committee.

## Author Contributions

BC and MR: conceptualization. YG, RL, CH, JB, CH-Z, AY, FZ, BC, AE, and MW-H: investigation. YG, JB, AY, BC, MK, MW-H, and MV: formal analysis. MR: wrote the manuscript. All authors revised the manuscript and approved the final version.

## Conflict of Interest

The authors declare that the research was conducted in the absence of any commercial or financial relationships that could be construed as a potential conflict of interest.
