# Supplementary material for: SOCS3 Expression by Thymic Stromal Cells Is Required for Normal T Cell Development
Source: Front Immunol. 2021 Mar 18;12:642173. doi: 10.3389/fimmu.2021.642173 (PMC8012910; doi:10.3389/fimmu.2021.642173)
Supplement: Supplementary file 1 [file Data_Sheet_1.DOCX]

Supplementary Material

# Supplementary Data

## Supplementary methods

The bead-bound proteins were reduced in 1 mM DTT at 60 degrees Celsius for 30 minutes. Next, samples were alkylated by incubation in the dark at room temperature in 5 mM chloro-acetamide for 20 minutes. After incubation the remaining chloro-acetamide was quenched by the addition of 4 mM DTT. Digestion was carried out by the addition of 1 µg Trypsin (sequencing grade modified, Pierce) and over-night incubation at 37°C. The next day the supernatant was collected and cleaned by a modified sp3 protocol [29]. Briefly, 10 µl Sera‐Mag SP3 bead mix (10 µg/µl) was added to the sample. Next, 100% acetonitrile was added to achieve a final concentration of >95%. Samples were pipette-mixed and incubated for 15 minutes at room temperature and then placed on a magnetic rack. The supernatant was aspirated and discarded and the beads were washed in 180 µl of acetonitrile. Samples were removed from the magnetic rack and beads were reconstituted in 15 µl of phase A (3% ACN 0.1% Formic Acid), followed by 1 minute of sonication. Then the beads were placed on a magnetic rack again and the supernatant was recovered and transferred to an MS-vial.

Q-Exactive Online LC-MS was performed using a Dionex Ultimate™ 3000 RSLCnano System coupled to a Q-Exactive mass spectrometer (Thermo Scientific). 7 uL was injected from each sample. Samples were trapped on a C18 guard desalting column (Acclaim PepMap 100, 75um x 2 cm, nanoViper, C18, 5 µm, 100 Å), and separated on a 50 cm long C18 column (Easy spray PepMap RSLC, C18, 2 um, 100Å, 75 umx15cm). The nano capillary solvent A was 95% water, 5%DMSO, 0.1% formic acid; and solvent B was 5% water, 5% DMSO, 95% acetonitrile, 0.1% formic acid. At a constant flow of 0.25 μl min^−1^, the curved gradient went from 6%B up to 43%B in 180 min, followed by a steep increase to 100% B in 5 min.

FTMS master scans with 60,000 resolution (and mass range 300-1500 m/z) were followed by data-dependent MS/MS (30 000 resolution) on the top 5 ions using higher energy collision dissociation (HCD) at 30% normalized collision energy. Precursors were isolated with a 2m/z window. Automatic gain control (AGC) targets were 1e6 for MS1 and 1e5 for MS2. Maximum injection times were 100ms for MS1 and MS2. The entire duty cycle lasted ~2.5s. Dynamic exclusion was used with 60s duration. Precursors with unassigned charge state or charge state 1 were excluded. An underfill ratio of 1% was used.

The MS raw files were searched using Sequest-Percolator or Target Decoy PSM Validator under the software platform Proteome Discoverer 1.4 (Thermo Scientific) against mouse Uniprot database and filtered to a 1% false discovery rate cut off.

# Supplementary Figures

## Supplementary Figure 1

##

### Reduced number of different thymocytes subpopulations in Δsocs3 thymi

**A**) Representative micrographs from haematoxylin and eosin-stained tissue sections from the lung and liver from WT and *Δsocs3* mice (n=4 per group analysed) 7 days after the last dose of Tm. Note that inflammatory infiltrates are not observed WT or *Δsocs3* tissues.The mean number of (**B**) DN, **(C)** DP, **(D)** CD4 SP and **(E)** CD8 SP thymocytes subpopulations ± SEM of *Δsocs3* and WT mice are depicted. **F)** The mean number of γδ T cells within DN *Δsocs3* and WT thymocytes ± SEM are shown. **G)** The mean number of DN thymocyte subpopulations in WT and *Δsocs3* thymocytes are depicted. Differences between groups are significant at **p<0.01 and *p<0.05 one-way ANOVA, n=5 animals per group.

**Supplementary figure 2**

###

### Reduced number of DPs thymocytes subpopulations in Δsocs3 bone marrow recipients

Radiation bone marrow (BM) chimeras were generated using WT and *Δsocs3* mice as recipients or donors. Sixty days after transplantation, mice were treated with Tm and sacrificed 7 days after the last dose. The mean number of *Δsocs3* and WT (**A**) DN, **(B)** DP, **(C)** CD4 SP and **(D)** CD8 SP thymocytes ± SEM are depicted. Differences between groups are significant at * p≤0.05, ** p≤0.01 unpaired one-way ANNOVA.  **E, F)** The mean % ± SEM of IL-7R+ **(E)** CD4 and **(F)** CD8 SP thymocytes in BM chimeras of *Δsocs3* and WT mice. Differences between groups are significant at * p≤0.05, ** p≤0.01 and *** p≤0.001, one-way ANNOVA and unpaired Student’s t test. The experiments were repeated twice with similar results. **G)** Histogram of IL7R expression on CD8 SP in *Δsocs3* and WT bone marrow chimeric mice. An isotype control is included in the overlay.

## Supplementary Figure 3

##

### SPs thymocytes display a more mature phenotype in Δsocs3 thymi

**A-H)** The mean MFI ± SEM and histograms of **(A-D)** Qa-2 and **(E-H)** CD24 expression in WT and *Δsocs3* CD4 (**A, B, E, F)** and CD8 SP **(C, D, G, H)** thymocytes are depicted. Differences between groups (n=5 animals per group) are significant at **p<0.01 Student’s *t* test. **I**) The mean frequencies of Foxp3+ cells within WT and *Δsocs3* CD4 SPs ± SEM (n= 6 per group) are depicted.

## Supplementary Figure 4

### T cell development in thymic organoids grafted in the kidney capsule

Photograph of the kidney showing the thymic graft **(A)** before and **(B)** after excision. **C)** Labelling of CD45.1+ thymocytes in the kidney graft resulting from CD45.2+ WT thymic implantation and **D)** representative dot plot analysis of the CD4 and CD8 expression in the CD45.1 gated thymocytes in a CD45.2 WT graft. **E, F)** The mean number of *socs3 lck cre* and *lck cre* DN, DP, CD4 SP and CD8 SP thymocytes ± SEM are depicted. Representative dot plots and, **F)** mean frequency ± SEM of β- and γδ TCR DN thymocytes in *Socs3^fl/fl^ cd4cre* and *cd4 cre* thymocytes ± SEM are shown. **G)** The dot plots of BrdU+ gated WT and *Δsocs3* lineage negative thymocyte populations labelled with CD4 and CD4, at 4 and 72h after BrdU administration are shown.

## Supplementary Figure 5


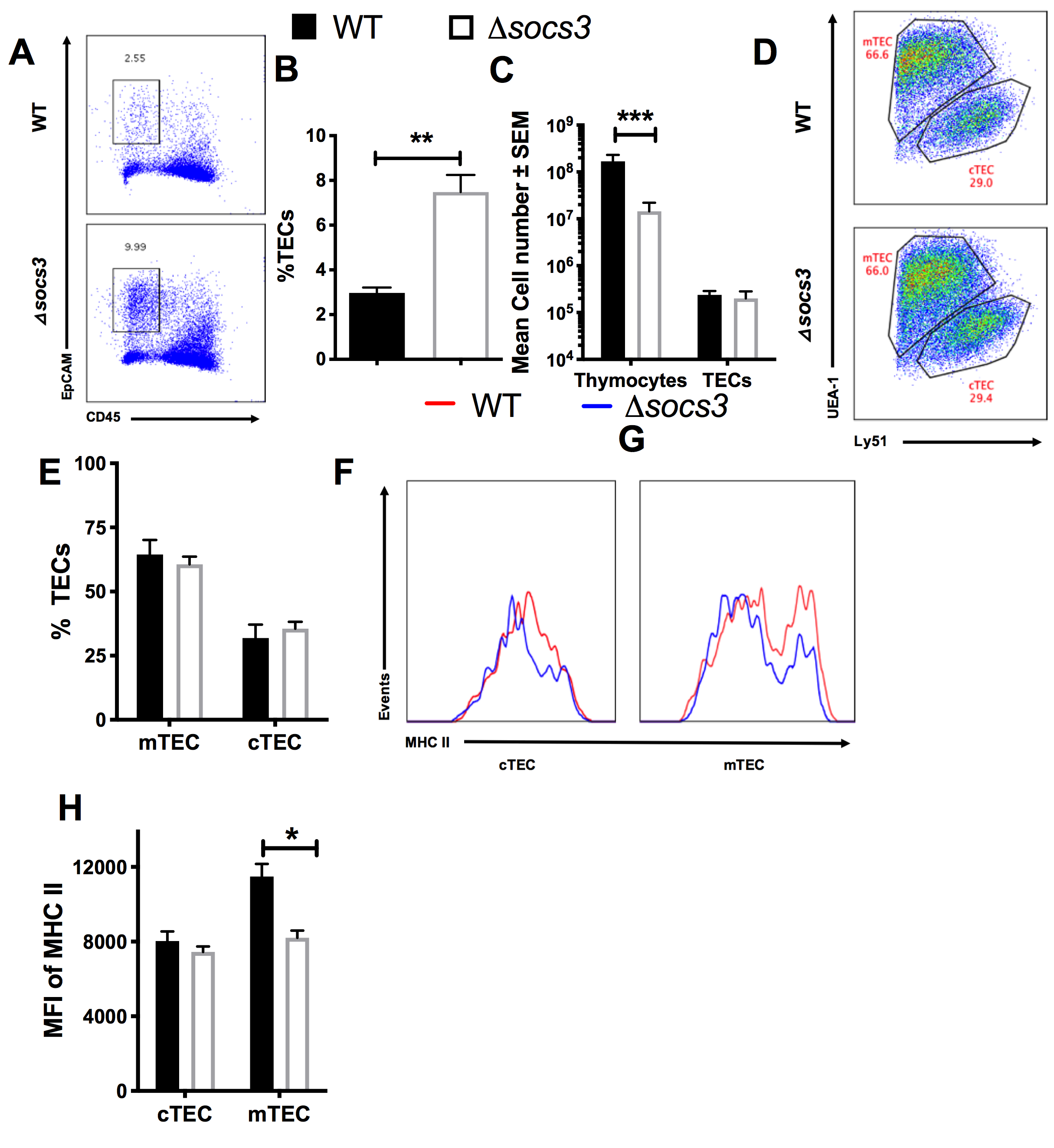


### Alterations in the thymus architecture in Δsocs3 mice

**A)** Representative dot plots showing CD45+ thymocytes and CD45-ve EpCam1+ TECs from WT and *Δsocs3* mice. **B)** The mean frequencies and (**C)** numbers of TECs and thymocytes ± SEM in WT and *Δsocs3* mice. Differences between groups (n=5 animals per group) are significant at **p<0.01 and ***p<0.001 Student’s *t* test. **D)** Representative dot plots and **(E)** the frequencies of mTECs and cTECs ± SEM. mTECs and cTECs were defined by the expression of UEA1 and Ly51 in CD45-EpCam1+ gated cells from WT and *Δsocs3* thymus. **F, G)** Representative histograms of MHC-II expression in **(F)** cTECs and **(G)** mTECs are shown. **H)** The mean frequency of mature (MHC-II^high^) WT and *Δsocs3* mTECs ± SEM is depicted. Differences between groups (n=5 animals per group) are significant at *p<0.05 Student’s *t* test.

## Supplementary Figure 6


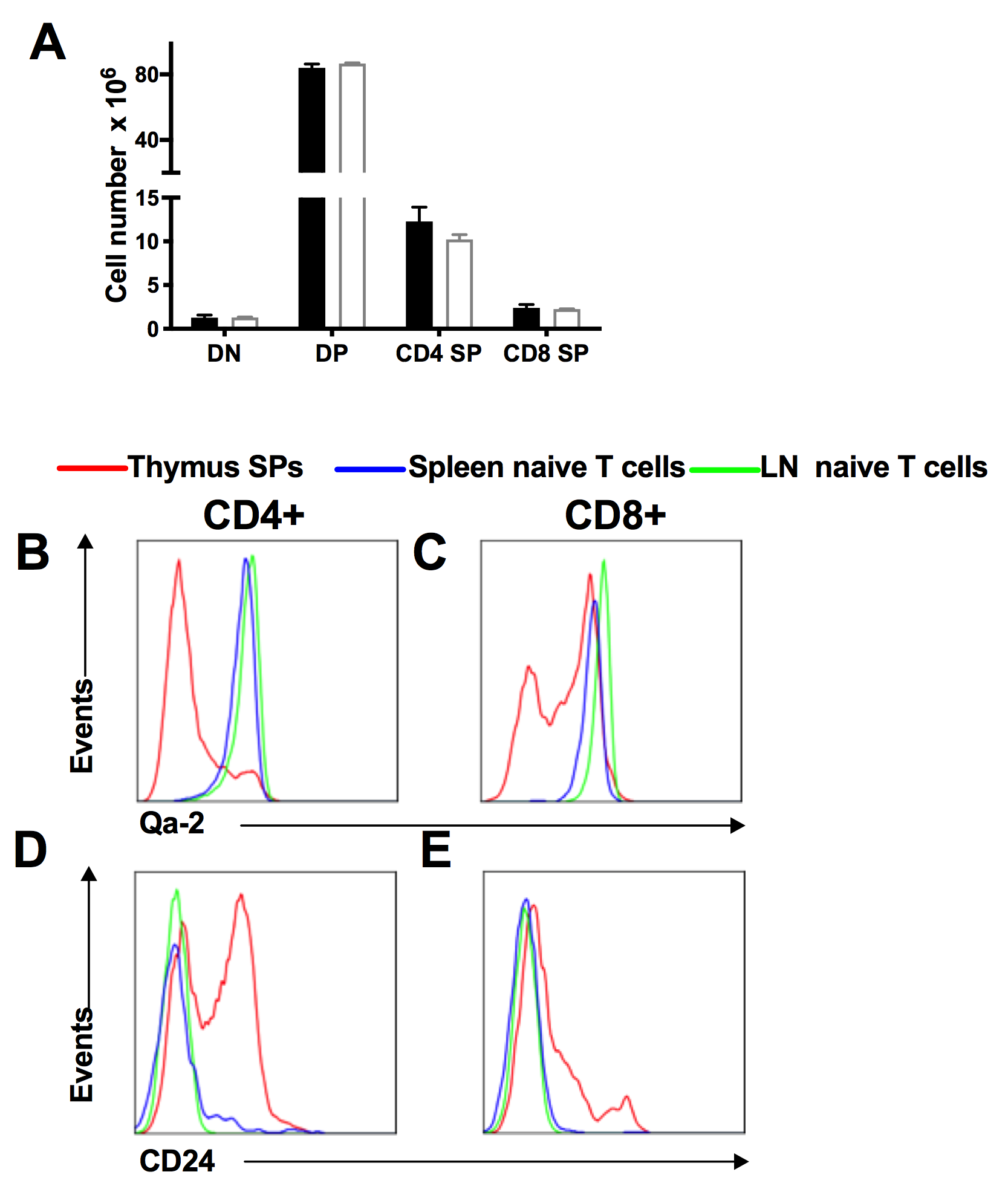


### Subpopulations in Δsocs3 and WT thymi transplanted with CD45.1BM cells.

**A)** The mean number ± SEM of different CD45.1+ thymocytes subpopulations in *Δsocs3* and WT recipients transplanted with CD45.1BM cells 60 days before sacrifice is depicted. Representative histograms depicting the expression of Qa-2 **(B, C)** and CD24 **(D, E)** in CD4 or CD8 SP thymocytes and (CD3+CD44-) naïve spleen and lymph node T cells from WT mice are shown.

# Supplementary tables

## Supplementary table 1

### KEEG gene ontology families regulated in Δsocs3 TECs

|  | **KEGG pathways** | **N# genes/ category** | **Observed** | **Expected** | **Ratio** | **p value** |  |
| --- | --- | --- | --- | --- | --- | --- | --- |
| Down-regulated | | Cytokine-cytokine receptor interaction | 245 | 17 | 3,1 | 5,49 | 9,6.10^-7^ |
|  |  | Systemic lupus erythematosus | 148 | 12 | 1,87 | 6,41 | 1,2.10^-7^ |
|  |  | Cell cycle | 127 | 10 | 1,61 | 6,23 | 9,3.10^-5^ |
|  |  | Asthma | 23 | 5 | 0,29 | 17,2 | 10^-4^ |
|  |  | Steroid hormone biosynthesis | 55 | 6 | 0,7 | 8,63 | 7.10^-4^ |
| Up-regulated | | TGF-beta signalling pathway | 84 | 8 | 0,97 | 8,26 | 3.10^-4^ |
|  |  | Cytokine-cytokine receptor interaction | 245 | 12 | 2,82 | 4,25 | 5.10^-4^ |
|  |  | Pathways in cancer | 322 | 14 | 3,71 | 3,77 | 5.10^-4^ |

## Supplementary Table 2

### KEEG database of gene families differentially expressed in Δsocs3 TECS

### 1. Up-regulated genes

A.TGF-β signaling pathway

| **Gene Symbol** | **Fold change** | **Gene Name** |
| --- | --- | --- |
| *Bmpr2* | 2,01 | bone morphogenetic protein receptor, type II (serine/threonine kinase) |
| *Thbs1* | 2,84 | thrombospondin 1 |
| *Id1* | 3,67 | inhibitor of DNA binding 1 |
| *Id3* | 2,68 | inhibitor of DNA binding 3 |
| *Thbs4* | 2,13 | thrombospondin 4 |
| *Fst* | 2,98 | follistatin |
| *Thbs2* | 2,57 | thrombospondin 2 |
| *Tgfbr1* | 2,08 | transforming growth factor, beta receptor I |

B. Cytokine receptor interaction

| **Gene Symbol** | **Fold change** | **Gene Name** |
| --- | --- | --- |
| *Bmpr2* | 2,01 | bone morphogenetic protein receptor, type II (serine/threonine kinase) |
| *Osmr* | 4,10 | oncostatin M receptor |
| *Tnfrsf1a* | 3,05 | tumor necrosis factor receptor superfamily, member 1a |
| *Cxcl14* | 2,55 | chemokine (C-X-C motif) ligand 14 |
| *Hgf* | 3,43 | hepatocyte growth factor |
| *Pdgfc* | 3,07 | platelet-derived growth factor, C polypeptide |
| *Il13ra1* | 2,00 | interleukin 13 receptor, alpha 1 |
| *Il2ra* | 2,24 | interleukin 2 receptor, alpha chain |
| *Il6st* | 3,30 | interleukin 6 signal transducer |
| *Lifr* | 2,25 | leukemia inhibitory factor receptor |
| *Il7* | 3,31 | interleukin 7 |
| *Tgfbr1* | 2,08 | transforming growth factor, beta receptor I |

### C. Pathways in cancer

| **Gene Symbol** | **Fold change** | **Gene Name** |
| --- | --- | --- |
| *Foxo1* | 2,15 | forkhead box O1 |
| *Ralgds* | 2,09 | ral guanine nucleotide dissociation stimulator |
| *Hgf* | 3,43 | hepatocyte growth factor |
| *Igf1r* | 2,68 | insulin-like growth factor I receptor |
| *Wnt4* | 2,48 | wingless-related MMTV integration site 4 |
| *Gli3* | 2,16 | GLI-Kruppel family member GLI3 |
| *Ar* | 2,09 | androgen receptor |
| *Fzd3* | 2,90 | frizzled homolog 3 (Drosophila) |
| *Mmp2* | 2,78 | matrix metallopeptidase 2 |
| *Fgf9* | 3,10 | fibroblast growth factor 9 |
| *Fgfr2* | 3,28 | fibroblast growth factor receptor 2 |
| *Cblb* | 2,39 | Casitas B-lineage lymphoma b |
| *Bcl2* | 2,30 | B cell leukemia/lymphoma 2 |
| *Tgfbr1* | 2,08 | transforming growth factor, beta receptor I |

### 2. Down-regulated genes

Cytokine receptor interaction

| **Gene Symbol** | **Fold change** | **Gene Name** |
| --- | --- | --- |
| *Ccl20* | -4,56 | chemokine (CCL20) |
| *Ccl9* | -4,53 | chemokine (CCL9) |
| *Tnfrsf11b* | -4,10 | osteoprotegerin |
| *Ccr4* | -3,99 | chemokine receptor (CCR4) |
| *Il5ra* | -3,67 | interleukin 5 receptor, alpha |
| *Ccl22* | -3,51 | chemokine (CCL22) |
| *Il2rg* | -3,21 | interleukin 2 receptor, gamma chain |
| *Cd30* | -2,93 | tumor necrosis factor receptor superfamily 8, (CD30) |
| *il23p19* | -2,86 | interleukin 23, alpha subunit p19 |
| *Il23r* | -2,49 | interleukin 23 receptor |
| *Tnf* | -2,42 | tumor necrosis factor |
| *Il12rb2* | -2,37 | interleukin 12R, beta 2 |
| *Cxcl9* | -2,36 | chemokine (CXCL9) |
| *Ccr7* | -2,16 | chemokine (C-C motif) receptor 7 |
| *Ccl6* | -2,15 | chemokine (CCL6) |
| *Il9* | -2,12 | interleukin 9 |
| *Cd40* | -2,07 | CD40 |
